# Construction and validation of a novel aging‐related gene signature and prognostic nomogram for predicting the overall survival in ovarian cancer

**DOI:** 10.1002/cam4.4404

**Published:** 2021-11-25

**Authors:** Lixiao Liu, Jinduo Zhao, Xuedan Du, Ye Zhao, Chengyang Zou, Heling Zhou, Wenfeng Li, Xiaojian Yan

**Affiliations:** ^1^ Department of Obstetrics and Gynecology The First Affiliated Hospital of Wenzhou Medical University Wenzhou China; ^2^ Department of Chemoradiation Oncology The First Affiliated Hospital of Wenzhou Medical University Wenzhou China; ^3^ Taizhou Hospital of Zhejiang Province affiliated to Wenzhou Medical University Wenzhou China

**Keywords:** aging, GEO, nomogram, ovarian cancer, signature, TCGA

## Abstract

**Background:**

Ovarian cancer (OC) is the most lethal gynecological malignancy. The objective of this study was to establish and validate an individual aging‐related gene signature and a clinical nomogram that can powerfully predict independently the overall survival rate of patients with ovarian cancer.

**Methods:**

Data on transcriptomic profile and relevant clinical information were retrieved from The Cancer Genome Atlas (TCGA) database as a training group, and the same data from three public Gene Expression Omnibus (GEO) databases as validation groups. Univariate Cox regression analysis, lasso regression analysis, and multiple multivariate Cox analysis were analyzed sequentially to select the genes to be included in the aging‐associated signature. A risk scoring model was established and verified, the predictive value of the model was evaluated, and a clinical nomogram was established.

**Results:**

We found eight genes that were most relevant to prognosis and constructed an eight‐mRNA signature. Based on the model, each OC patient's risk score was able to be calculated and patients were split into groups of low and high risks with a distinct outcome. Survival analysis confirmed that the outcome of patients in the high‐risk group was dramatically shorter than that of those in the low‐risk group, and the eight‐mRNA signature can be considered as a powerful and independent predictor that could predict the outcome of OC patient. Additionally, the risk score and age can be used to construct a clinical nomogram as a simpler tool for predicting prognosis. We also explored the association between the risk score and immunity and drug sensitivity.

**Conclusion:**

This study suggested that the aging‐related gene signature could be used as an intervention point and latent prognostic predictor in OC, which may provide new perceptions for postoperative treatment strategies.

## Graphical abstract

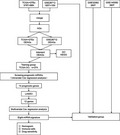

## 1 INTRODUCTION

Ovarian cancer (OC) is the most lethal gynecological malignancy in females, with a high rate of recurrence.^1^ According to 2020 global cancer statistics, more than 310,000 new cases and more than 200,000 deaths worldwide were reported.^2^ As early OC is asymptomatic and the fact that the late symptoms of OC are nonspecific, and there is no effective screening method for early OC, the disease is often already at an advanced and incurable stage when it is diagnosed.^3^, ^4^, ^5^, ^6^ For decades, the treatment of OC has largely involved surgery and platinum chemotherapy.^7^ The survival rate at 5 years for stage I OC has exceeded 90%, while that of stage III or IV female patients is less than 25%.^3^, ^8^, ^9^, ^10^ At present, transvaginal ultrasound and measurement of biomarkers such as CA125 are the two most commonly used screening methods for OC, but according to clinical trials, these two screening methods do not increase the survival rate of patients.^11^, ^12^, ^13^ Hence, it is of major significance to identify new biological indicators for diagnosis and prognosis.

Due to population growth and aging, the incidence of cancer will continue to increase over the next 20 years.^2^ Aging and cancer have become inseparable.^14^ Age is one of the strongest and most frequently studied risk factors for OC incidence and mortality.^15^, ^16^, ^17^ Meanwhile, the changes in gene expression in tumor tissues are stably correlated with the prognosis of cancer.^18^, ^19^, ^20^ Therefore, by studying the expression level of aging‐related genes in tumor tissue, we can identify the subgroup of patients with worse prognosis earlier and carry out corresponding clinical treatment as soon as possible to confer a survival advantage in patients. Recent studies have revealed a model constructed using age‐related genes to aid in the prediction of outcome in colorectal cancer patients.^21^ In glioma, aging has been found to be the strongest risk factor for disease progression, and the survival of glioma patients can be predicted by analyzing brain aging.^22^ However, markers for aging‐related gene in predicting the survival rate of OC patients have not yet been established.

In this study, we integrated The Cancer Genome Atlas (TCGA) and Gene Expression Omnibus (GEO) databases, developed and validated personalized prognostic markers based on aging‐related genes, and constructed a nomogram that validated the stability and sensitivity of the model. Moreover, we comprehensively analyzed patient clinical information, immune cell infiltration, and sensitivity to drugs to increase the accuracy rating of the overall prediction.

## 2 METHOD

### 2.1 Data collection and processing

The RNA‐Seq data and relevant clinical data of 379 OC patients were retrieved from the TCGA (https://portal.gdc.cancer.gov/) database as the training set. The inclusion criteria for OC patients were as follows: (1) only patients with primary ovarian cancer were selected; (2) patients had complete RNA sequencing data and clinicopathological parameters available; (3) patients were followed up with for at least 30 days; and (4) overall survival (OS) was the primary endpoint. The full name of GEO database (https://www.ncbi.nlm.nih.gov/geo/) is GENE EXPRESSION OMNIBUS. It contains high‐throughput gene expression data submitted by research institutions around the world, that is to say as long as it is currently published papers, the data for the gene expression tests covered in the paper can be found in this database. We searched for “ovarian cancer AND survival” in the GEO database, and the data sets with complete survival data and the sample sizes in the data sets are greater than 100 was used as the validation sets. Thereupon, the mRNA expression and clinical data of 185 cases of ovarian cancer and 10 normal ovarian tissues from the GSE26712 data set were searched and downloaded as independent validation set 1 from the GEO database. The mRNA expression and clinical data of 260 cases of ovarian cancer from the GSE32062 data set were searched and downloaded as independent validation set 2. The mRNA expression and clinical data of 380 cases of ovarian cancer from the GSE140082 data set were searched and downloaded as independent validation set 3. In addition, Genome Tissue Expression (GTEx) (https://xenabrowser.net/) was used to collect gene expression data from 88 normal ovarian tissues. The downloaded data were standardized by the contributors. To better screen the differentially expressed genes, batch effects correction was performed using the "sva" package of R software to merge the RNA‐Seq data from the TCGA, GTEx, and GSE26712 data sets to eliminate the batch effect. In view of this operation, GSE26712 is used as an internal verification set, while GSE32062 and GSE140082 are used as an external verification set.

### 2.2 Screening of aging‐related genes

The complete list of aging‐related genes was downloaded from the Human Aging Genome Resource (HAGR, http://genomics.senescence.info/genes/), which includes 307 human aging genes in all (Table S1). The aging‐related genes (AGs) were determined by intersection of the merged genes and the aging genes.

### 2.3 Identification of differentially expressed AGs

The "limma" package was used to calculate the differentially expressed AGs (DEAGs) in the TCGA and GSE26712 data sets, and a heatmap and volcano map were drawn to visualize them. Then, a Venn diagram was drawn using the "venndiagram" package, and the genes co‐upregulated or co‐downregulated in the TCGA and GSE26712 data sets were selected as candidate genes for subsequent analysis.

### 2.4 Bioinformatic analysis

Gene Ontology (GO) enrichment analysis was used to explore the potential biological process (BP), molecular function (MF), and cellular component (CC) of candidate genes, and Kyoto Encyclopedia of Genes and Genes (KEGG) analysis was used to identify the signaling pathways of all the DEAGs. All analyses were conducted using the cluster profiler package.

### 2.5 Construction of an 8‐mRNA prognostic signature based on aging‐related genes

We use the TCGA database as the test set to construct the 8‐mRNA prognostic signature. Selecting *p* < 0.05 as the filter condition and using the R package "survival" to conduct a univariate Cox regression analysis on the TCGA training set to identify genes related to prognosis. Next, using the "glmnet" package, the least absolute shrinkage and selection operator (Lasso) regression analysis was used to remove collinearity of DEAGs and minimize overfitting of the model. Afterward, multivariate Cox regression was used to identify final DEAGs involved in the final signature and to obtain a correlation coefficient to construct a prognostic risk score formula. The formula was as follows: Risk score = β gene(1) × expression gene(1) + β gene(2) × expression gene(2) + ···+ β gene(n) × expression gene(n). Patients were dichotomized into two subgroups by the patient's cut‐off value of the median risk score, patients with a risk score higher than the median were classified as the high‐risk group, and vice versa for the low‐risk group. The Kaplan–Meier (K–M) curves were constructed with the “tidyverse” package and “survminer” package to compare OS rate differences between the high‐ and low‐risk groups, and the receiver operating characteristic curve (ROC) curves and the calibration curves were compared to investigate the accuracy, sensitivity, and specificity of the model.

### 2.6 Construction of a nomogram

Cox proportional regressions were performed for univariate and multivariate on age, grade, stage, and other clinicopathological parameters and the risk score to evaluate prognosis‐related independent factors for each parameter. The “rms” package and “survival” package were used to construct a nomogram together with the results of multivariate Cox regression, and 3‐, 5‐, and 7‐year correction curves were drawn to assess the predictive stability of the nomogram.

### 2.7 Correlation analysis of risk value and immune cells

We downloaded the immune cell infiltration table of the TCGA tumors from TIMER2.0 (http://timer.comp‐genomics.org/), and “limma,” “scales,” “ggplot2,” “ggtext,” and “ggpubr” were used to analyze the correlation between the risk score and immune cells. The immune cell differences between the high‐ and low‐risk groups were compared.

### 2.8 Drug sensitivity analysis

We used the Bioconductor package (“car,” “ridge,” “preprocess core,” “generater,” and “SVA”) to predict the sensitivity of high‐ and low‐risk groups to platinum drugs through IC50 and drew box plots.

### 2.9 External verification of eight‐mRNA signature

In the validation phase, the signature was validated in the internal validation cohort (GSE26712 data set) and in the external validation cohorts (GSE140082, and GSE32062 data sets). Risk scores were determined in the validation samples based on the above equation and patients were dichotomized into high‐ and low‐risk groups by their optimal cut‐off point. The K–M curve was drawn and the log‐rank test was utilized to visually compare the difference in OS between the two groups, and the ROC curve was drawn to evaluate the stability of the eight‐mRNA signature.

### 2.10 The expression of genes was verified by qPCR

Total RNA was extracted from OC samples and normal renal tissue samples using TRIzol reagent (Thermo Fisher Scientific, Waltham, MA, USA). Single‐stranded cDNA was synthesized from 1 µg of total RNA using the PrimeScript RT Reagent Kit with gDNA Eraser (Takara Biotechnology Co. Ltd., Dalian, China). Reverse transcription quantitative PCR was applied to explore the mRNA expression of the hub genes using a 7500 PCR system (Thermo Fisher Scientific). The following cycling conditions were adopted: 95°C for 5 min, followed by 40 cycles of 95°C for 10 s and 60°C for 30 s. The qPCR assays were performed for each sample in a reaction volume of 10 µL. The relative expression of genes in our signature was calculated using the 2‐Ct method. The following qPCR primer sequences were used: β‐actin forward primer 5′‐CATGTACGTTGCTATCCAGGC‐3′; β‐actin reverse primer 5′‐CTCCTTAATGTCACGCACGAT‐3′; JAK2 forward primer5′‐TCTGGGGAGTATGTTGCAGAA‐3′; JAK2 reverse primer5′‐AGACATGGTTGGGTGGATACC‐3′; IL2RG forward primer5′‐GTGCAGCCACTATCTATTCTCTG‐3′; IL2RG reverse primer5′‐GTGAAGTGTTAGGTTCTCTGGAG‐3′; EEF1E1 forward primer5′‐CCCTGGGACTGAGTAAGGGG‐3′; EEF1E1 reverse primer5′‐GTTGGCTTGCTTGACTAGATGA‐3′; UBB forward primer5′‐GGTCCTGCGTCTGAGAGGT‐3′; UBB reverse primer 5′‐GGCCTTCACATTTTCGATGGT‐3′; EPS8 forward primer5′‐TGAATGGCTACGGATCATCACC‐3′; EPS8 reverse primer5′‐CACTGTCCCGTGCATAATTCT‐3′; FOXO1 forward primer5′‐TCGTCATAATCTGTCCCTACACA‐3′; FOXO1 reverse primer5′‐CGGCTTCGGCTCTTAGCAAA‐3′; STAT5A forward primer 5′‐GCAGAGTCCGTGACAGAGG‐3′; STAT5A reverse primer5′‐CCACAGGTAGGGACAGAGTCT‐3′; PAPPA forward primer5′‐ACAAAGACCCACGCTACTTTTT‐3′; and PAPPA reverse primer5′‐CATGAACTGCCCATCATAGGTG‐3′.

### 2.11 Statistical analysis

All analyses were performed using R version 4.0.5. Univariate COX analyses were used to calculate the hazard ratio (HR) and 95% confidence intervals (CIs) to identify genes associated with OS. Multivariate Cox regression analysis was used for factors significantly associated with OS in the univariate analysis. The Kaplan–Meier method was used to compare the OS time of patients. The prediction accuracy of the risk characteristics and the calibration for the nomogram were determined by the ROC curve. All experimental data were analyzed using GraphPad Prism 5 Software (GraphPad Software Inc., La Jolla, CA, USA), and *t*‐test was used to test the differences between tumor and normal tissue in PCR. *p* < 0.05 was deemed to be significant unless indicated otherwise.

## 3 RESULT

### 3.1 Clinical information about patients included in the study

**FIGURE 1:**
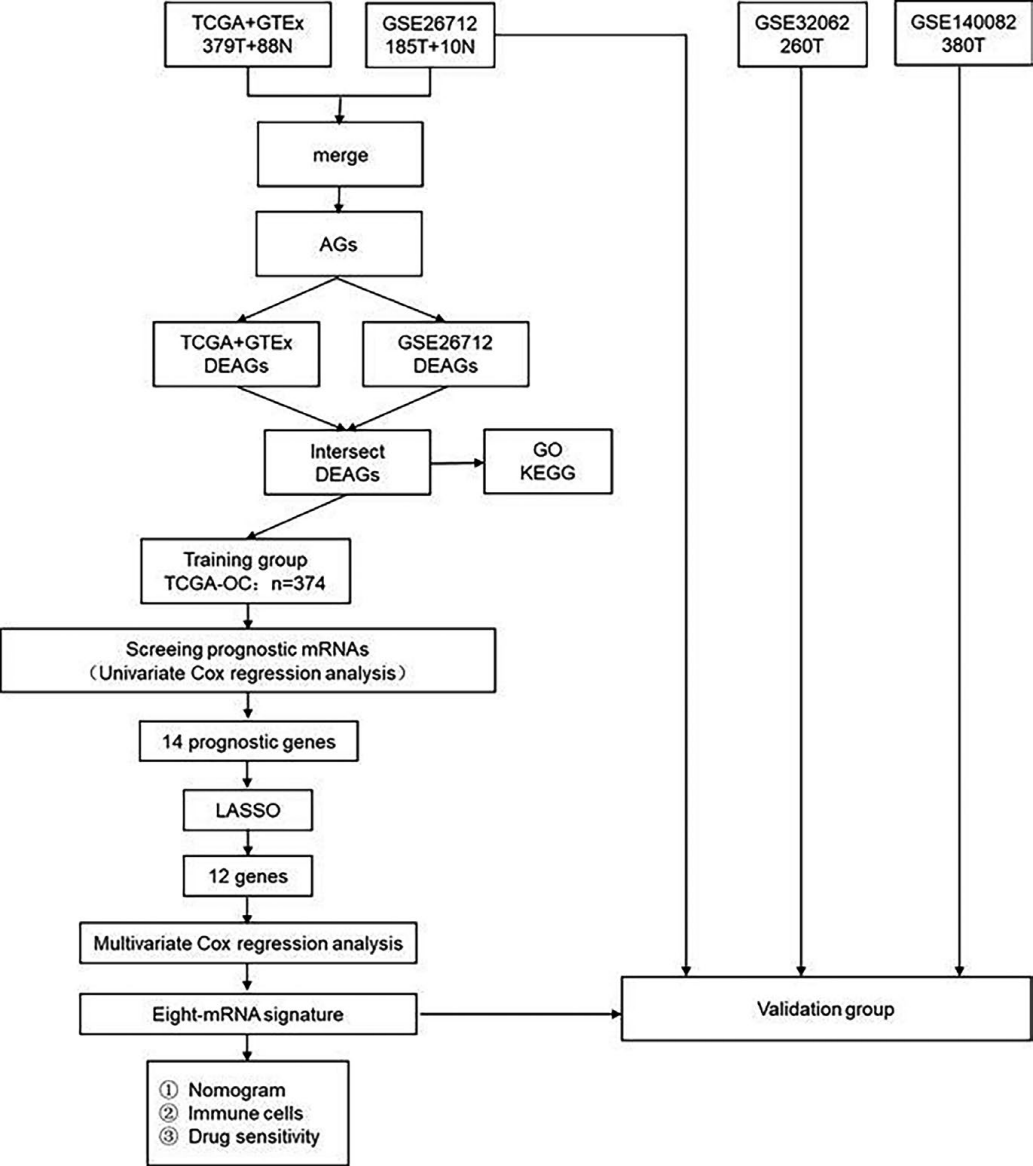
The flow chart for constructing and verifying the eight‐mRNA signature

Figure 1 shows the flow chart for constructing and verifying the eight‐mRNA signature. A total of 374 OC patients in the TCGA database were included in the training group, 185 OC patients in the GSE26712 data set were included in internal validation cohort, and 260 OC patients in the GSE32062 data set and 380 OC patients in the GSE140082 data set were included in external validation cohorts. The baseline clinical characteristics of the training group and validation groups are shown in Table 1.

**TABLE 1:** Clinicopathological characteristics of the patients included in the training group and validation groups

Variables	Training group TCGA No.	Validation group GSE26712 No.	Validation group GSE32062 No.	Validation group GSE140082 No.
No. of patients	374	185	260	380
Age				
<65	243	–	–	257
>=65	131	–	–	123
Vital status				
Alive	145	56	139	284
Dead	229	129	121	97
FIGO stage				
Stage I	1	–	–	20
Stage II	22	–	–	31
Stage III	291	–	204	266
Stage IV	57	–	56	62
Unknown	3	–	–	–
Grade				
G1	1	–	–	–
G2	42	–	131	–
G3	320	–	129	–
G4	1	–	–	–
Unknown	10	–	–	–
Average follow‐up time(year)	3.27	3.91	3.73	2.07

### 3.2 Screening of DEAGs

**FIGURE 2:**
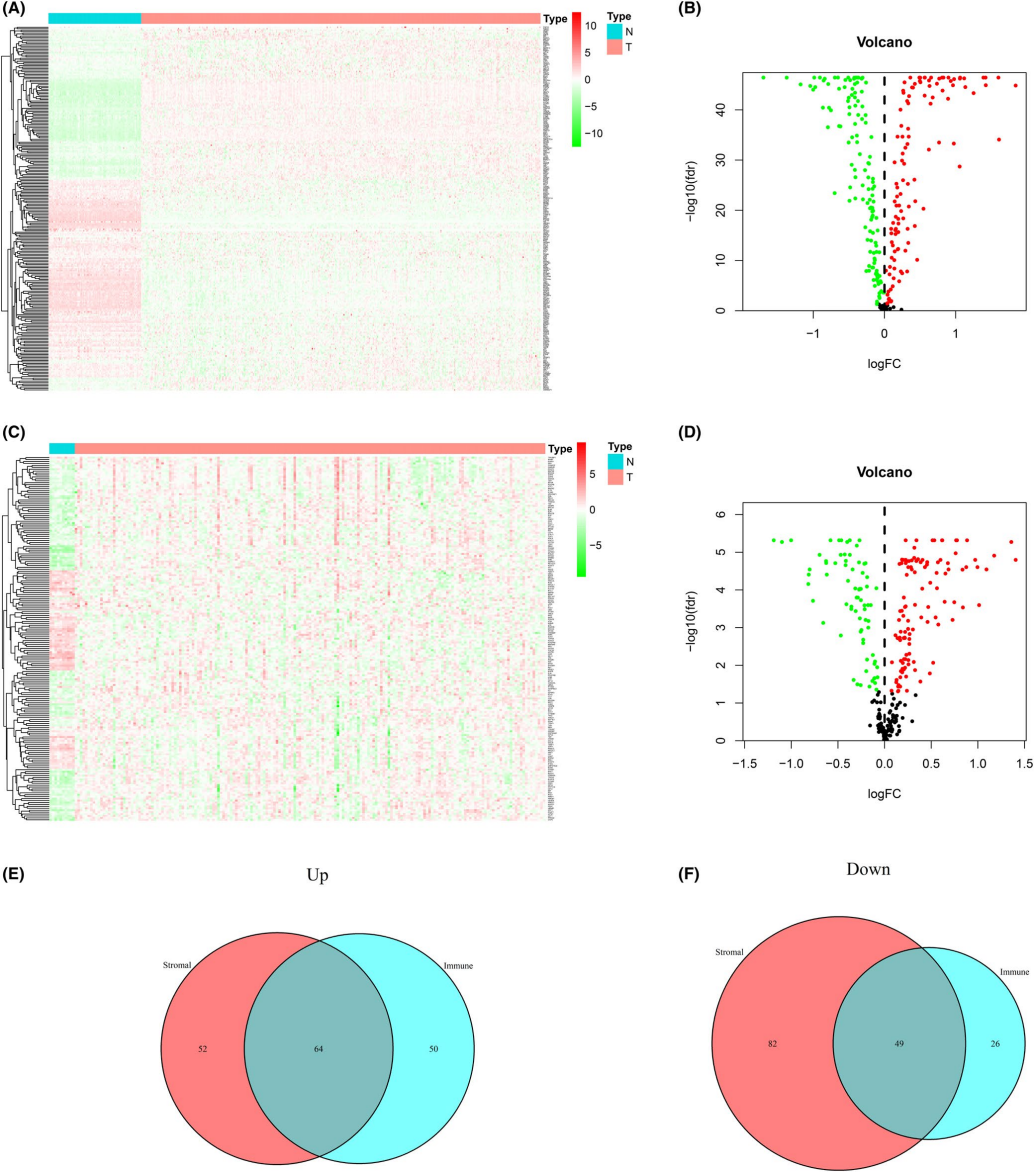
Heatmaps, volcano maps, and Venn diagrams of differentially expressed genes between normal tissue and ovarian cancer. (A) Heatmap and (B) volcano map demonstrating the 247 DEAGs of TCGA+GTEx. (C)Heatmap and (D) volcano map demonstrating the 189 DEAGs of GSE26712. (E) Jointly upregulated DEAGs. (F) Jointly downregulated DEAGs

Since both tumor samples and normal tissue samples were present in the TCGA, GTEX, and GSE26712 data sets, to better screen for common differential genes, we merged the RNA‐Seq data of the TCGA, GTEX, and GSE26712 data sets and eliminated the batch effect. Then, the merged transcriptome matrix was crossed with 307 aging genes to obtain the expression of 285 aging‐related genes. Among these 285 genes, 247 DEAGs (including 131 downregulated genes and 116 upregulated genes) were obtained by comparing the expression of the AGs in the TCGA tumor samples with that of the AGs in the GTEX normal samples. The 247 DEAGs are illustrated in the heatmap (Figure 2A) and volcano map (Figure 2B). Additionally, we compared the expression of AGs in normal tissues and tumor tissues in the GSE26712 data set, extracted 189 DEAGs (including 75 downregulated genes and 114 upregulated genes), and presented them in a heatmap (Figure 2C) and volcano map (Figure 2D). After identifying the intersection of the two sets of DEAGs, we obtained 64 jointly upregulated DEAGs (Figure 2E) and 49 jointly downregulated DEAGs (Figure 2F). These 113 DEAGs were used as candidate genes for constructing the signature.

### 3.3 Enrichment analysis of DEAGs function

**FIGURE 3:**
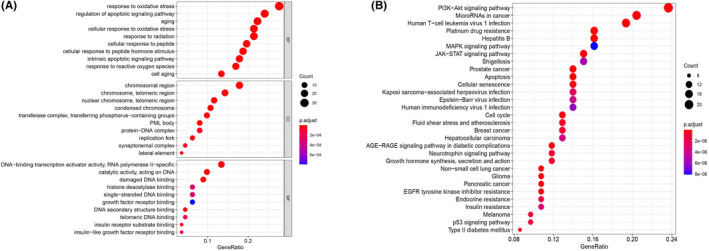
Functional enrichment analysis of DEAGs. (A) GO enrichment analysis; (B) KEGG pathway enrichment analysis

To analyze the potential biological functions of the DEAGs, we used the cluster Profiler package of R software for functional enrichment analysis. The GO analysis results (Figure 3A) showed that the top 10 enrichment scatter plots of the 113 DEAGs in BPs were concentrated in response to oxidative stress, regulation of apoptotic signaling pathway, aging, and cellular response to oxidative stress. The top 10 enrichment scatter plots of the CCs are concentrated in chromosomal regions, chromosomes, and telomeric regions. The enrichment scatter plot of the MFs mainly consists of DNA‐binding transcription activator activity and RNA polymerase II specificity. The results of KEGG analysis showed (Figure 3B) that the PI3K‐Akt signaling pathway and microRNAs in the cancer pathway were the most obvious regions of DEAG enrichment. Enrichment analysis showed that DEAGs are closely related to aging, oxidative stress, chromosomal changes, and cancer.

### 3.4 Screening of prognostic DEAGs and construction of an eight‐mRNA signature

**FIGURE 4:**
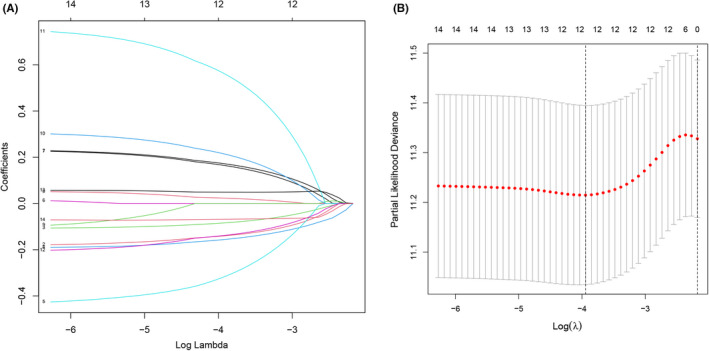
Lasso regression. (A) The variation trajectory of each independent variable. The logarithm of the independent variable lambda was taken as the horizontal axis, and the coefficient of the independent variable was taken as the vertical axis. (B) Confidence intervals for each phase for each lambda, the vertical black dotted lines defined the optimal values of lambda, which provides the best fit

**FIGURE 5:**
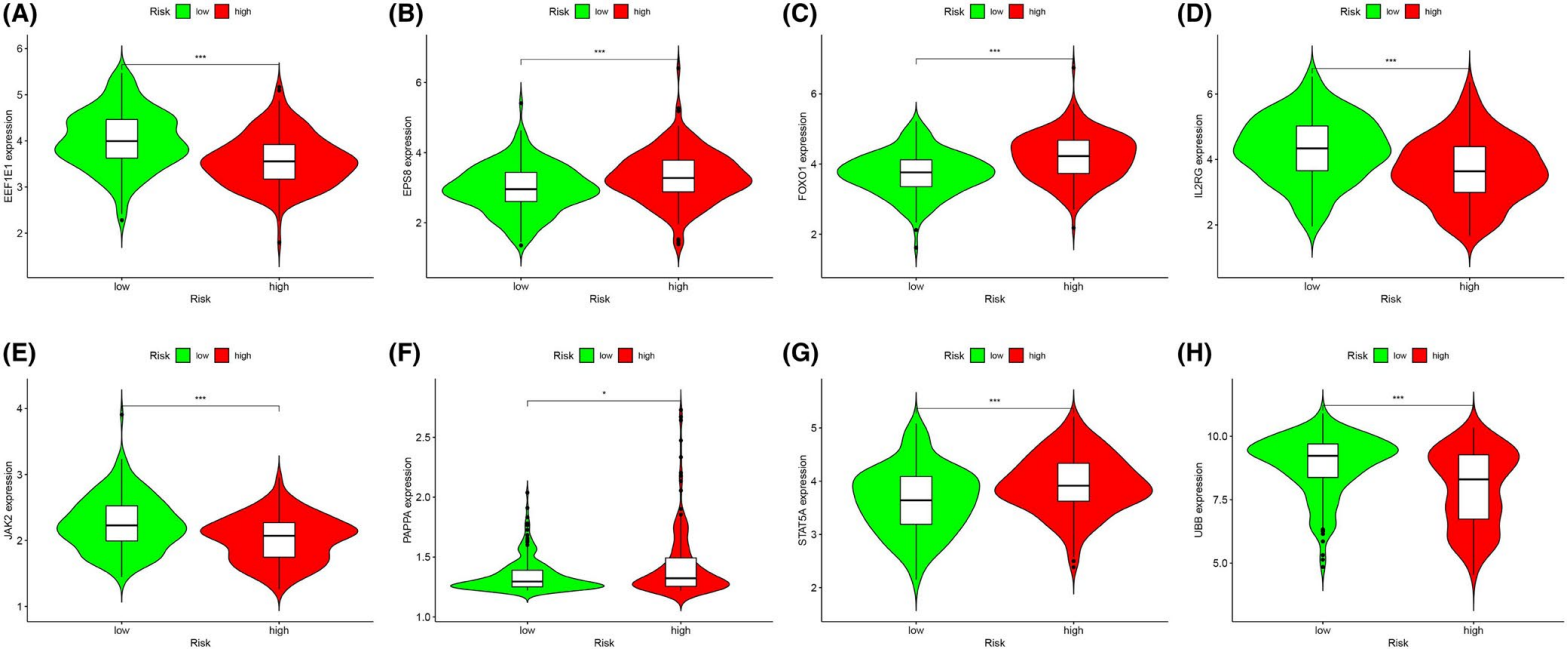
The violin diagram of the expression levels of eight genes in the training group. (A) EEF1E1, (B) EPS8, (C) FOXO1, (D) IL2RG, (E) JAK2, (F) PAPPA, (G) STAT5A, and (H) UBB

In the process of screening for biomarkers related to prognosis, we performed univariate Cox analysis on the 113 DEAGs that were jointly upregulated or downregulated in the TCGA training group. As a result, 14 DEAGs were significantly correlated with the OS rate of the 374 OC patients (*p* < 0.05). Subsequently, the candidate genes were reduced to 12 using the Lasso algorithm to prevent gene overfitting (Figure 4A,B). Finally, through multivariate Cox analysis, we obtained the following eight DEAGs that are most relevant to the prognosis of OC for constructing an aging gene‐related risk scoring signature: JAK2, IL2RG, EEF1E1, UBB, EPS8, FOXO1, STAT5A, and PAPPA. The details of these eight genes are shown in Table 2. The prognostic risk score for each patient was imputed below: Risk score = (−0.457984 × JAK2) + (−0.205973 × IL2RG) + (−0.184204 × EEF1E1) + (−0.098419 × UBB) + (0.227572 × EPS8) + (0.272872 × FOXO1) + (0.349501 × STAT5A) + (0.754430 × PAPPA). A violin diagram of the distribution of these eight gene expression in the training group is shown in Figure 5A–H. In tumor tissues, four protective genes (JAK2, IL2RG, EEF1E1, and UBB) showed relatively low expression in the high‐risk group; in contrast, the expression of four risk genes (EPS8, FOXO1, STAT5A, and PAPPA) was more highly in the high‐risk group than that in the low‐risk group.

**TABLE 2:** 8‐gene signature selected by multivariate Cox regression

Name	Coefficient	Type	Down/upregulated	HR	95%CI	*p* value
JAK2	−0.45798	Protective	Down	0.63	0.40–1.00	0.0497283
IL2RG	−0.20597	Protective	Up	0.81	0.69–0.96	0.0158012
EEF1E1	−0.1842	Protective	Up	0.83	0.65–1.06	0.1319088
UBB	−0.09842	Protective	Down	0.91	0.83–0.99	0.0382452
EPS8	0.227572	Risky	Down	1.26	1.03–1.54	0.02776
FOXO1	0.272872	Risky	Down	1.31	1.07–1.62	0.0098715
STAT5A	0.349501	Risky	Down	1.42	1.11–1.81	0.0050247
PAPPA	0.75443	Risky	Down	2.13	1.19–3.79	0.0103691

### 3.5 Identification and verification of the survival predictive power of the eight‐mRNA signature

**FIGURE 6:**
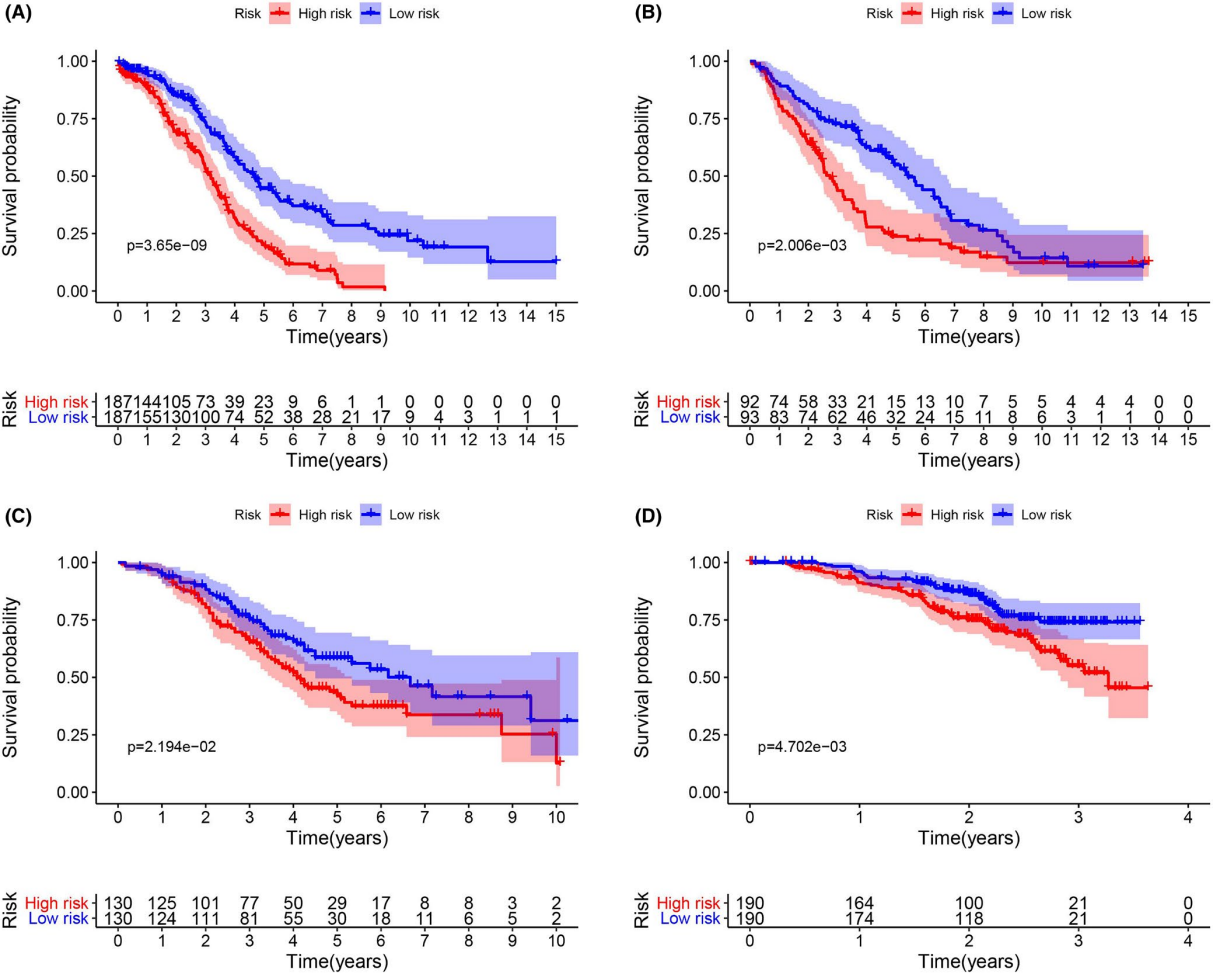
Identification and verification of the predictive eight‐mRNA signature. K–M curves in the training group (A), GSE26712 data group (B), GSE32062 data group (C), and GSE140082 data group (D)

**FIGURE 7:**
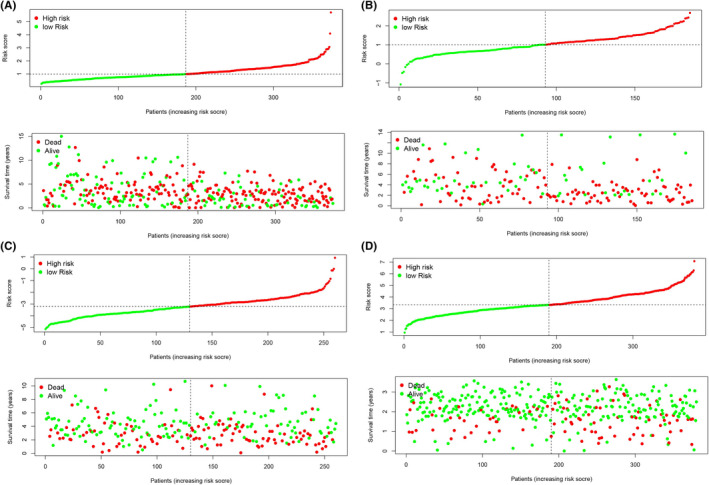
The distribution of risk score, survival time, and survival status in the training group (A), GSE26712 data group (B), GSE32062 data group (C), and GSE140082 data group (D)

**FIGURE 8:**
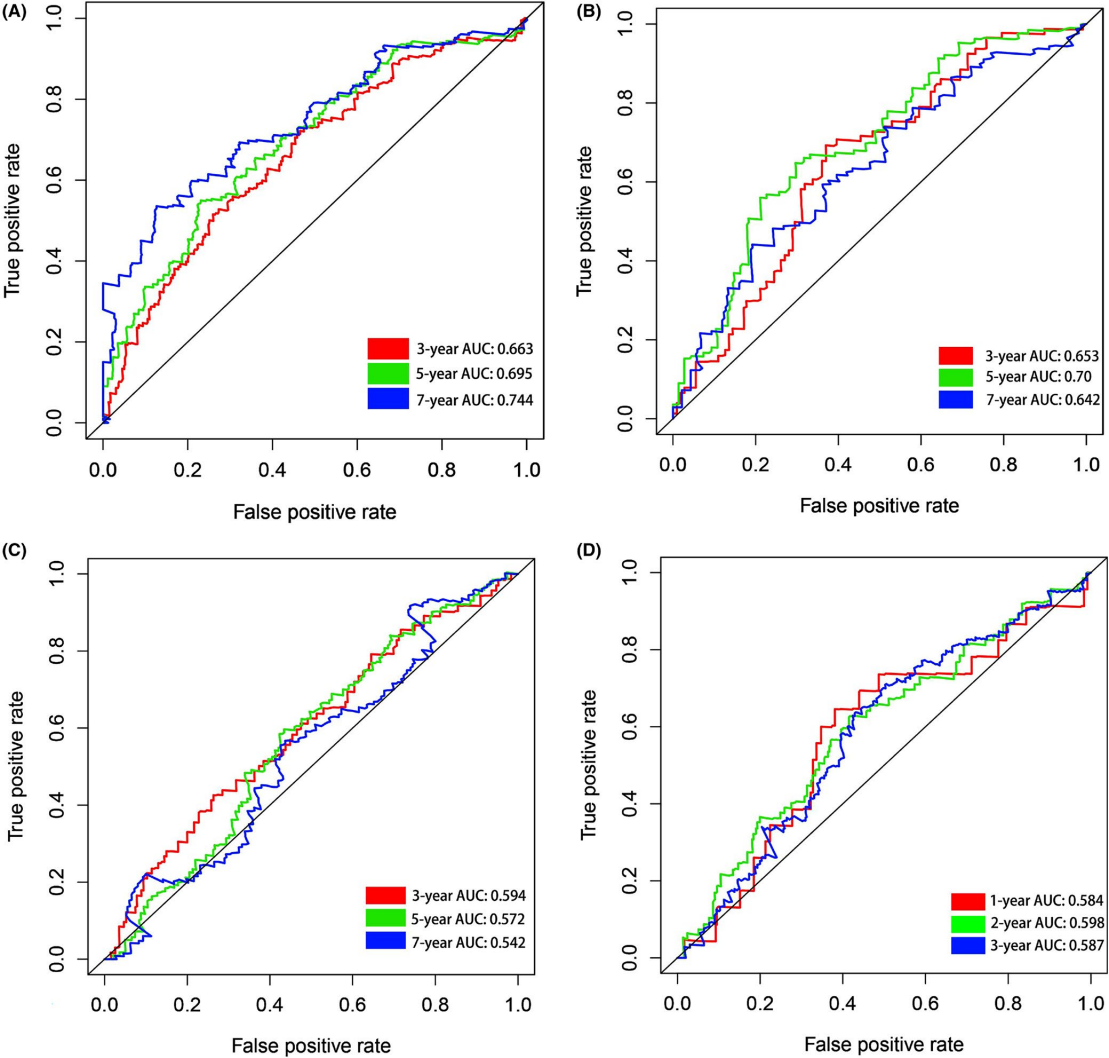
The ROC curve over time in the training group (A), GSE26712 data group (B), GSE32062 data group (C), and GSE140082 data group (D)

The risk scores of the TCGA training group (*n* = 374), GSE26712 data group (*n* = 185), GSE32062 data group (*n* = 260), and GSE140082 data group (*n* = 380) were calculated by the same risk score formula. Using their own median score as the cut‐off value, patients, and the K–M curve and ROC curve related to the risk model were drawn accordingly. Survival analysis revealed that patients in the high‐risk group of both training group and the validation groups exhibited a significantly poorer OS rate than the low‐risk group (Figure 6A–D). The distribution of risk score, survival time, and survival status is plotted in Figure 7. The results showed that the mortality of patients was significantly elevated with the survival time gradually decreased as the progressively increasing risk score (Figure 7A–D). In the TCGA test set, ROC values of 3‐, 5‐, and 7‐year were 0.663, 0.695, and 0.744, respectively; in the GSE26712 data group, ROC values of 3‐, 5‐, and 7‐year were 0.653, 0.70, and 0.642, respectively; In GSE32062 data group, ROC values of 3‐, 5‐, and 7‐year were 0.594, 0.572, and 0.542, respectively; In GSE140082 data group, ROC values of 3‐, 5‐, and 7‐year were 0.584, 0.598, and 0.587, respectively. The ROC curve over time showed that the eight‐mRNA signature had a promising and a broad applicability in predicting the OS rate of OC patients (Figure 8A–D). Then, we drew the calibration curve of each group for 3 years, and the results proved that the predicted value had good coincidence with the actual value, and proved the good stability of our model (Figure S1A–D).

### 3.6 Eight‐mRNA signature was identified as an independent prognostic marker for OC patients

To investigate whether the any of the variables have independent prognostic significance of the OS rate of patients with OC, we conducted additional univariate Cox analysis and multivariate Cox analysis on clinical factors such as risk score, age, grade, and stage of the training group. Univariate Cox analysis showed that risk score and age were significantly related the OS rate of OC patients. The significant factors in the univariate Cox analysis were included in the multivariate Cox analysis. After the multivariate Cox analysis, the risk score and age remained as independent prognostic factors (Table 3). Taken together, these results show that the combined 8‐mRNA risk model is an excellent prognostic marker independent of other clinical features.

**TABLE 3:** Univariate and multivariate Cox regression analyses of the gene signature

	Univariate analysis			Multivariate analysis		
Feature	HR	95%CI	*p* value	HR	95%CI	*p* value
Risk	2.220	1.69–2.91	8.47E−09	2.164	1.65–2.84	2.87E−08
Age	1.020	1.01–1.03	0.001131	1.018	1.01–1.03	0.004326
Grade	1.194	0.80–1.79	0.38942	–	–	–
Stage	2.085	0.93–4.70	0.076316	–	–	–

### 3.7 Construct a nomogram

**FIGURE 9:**
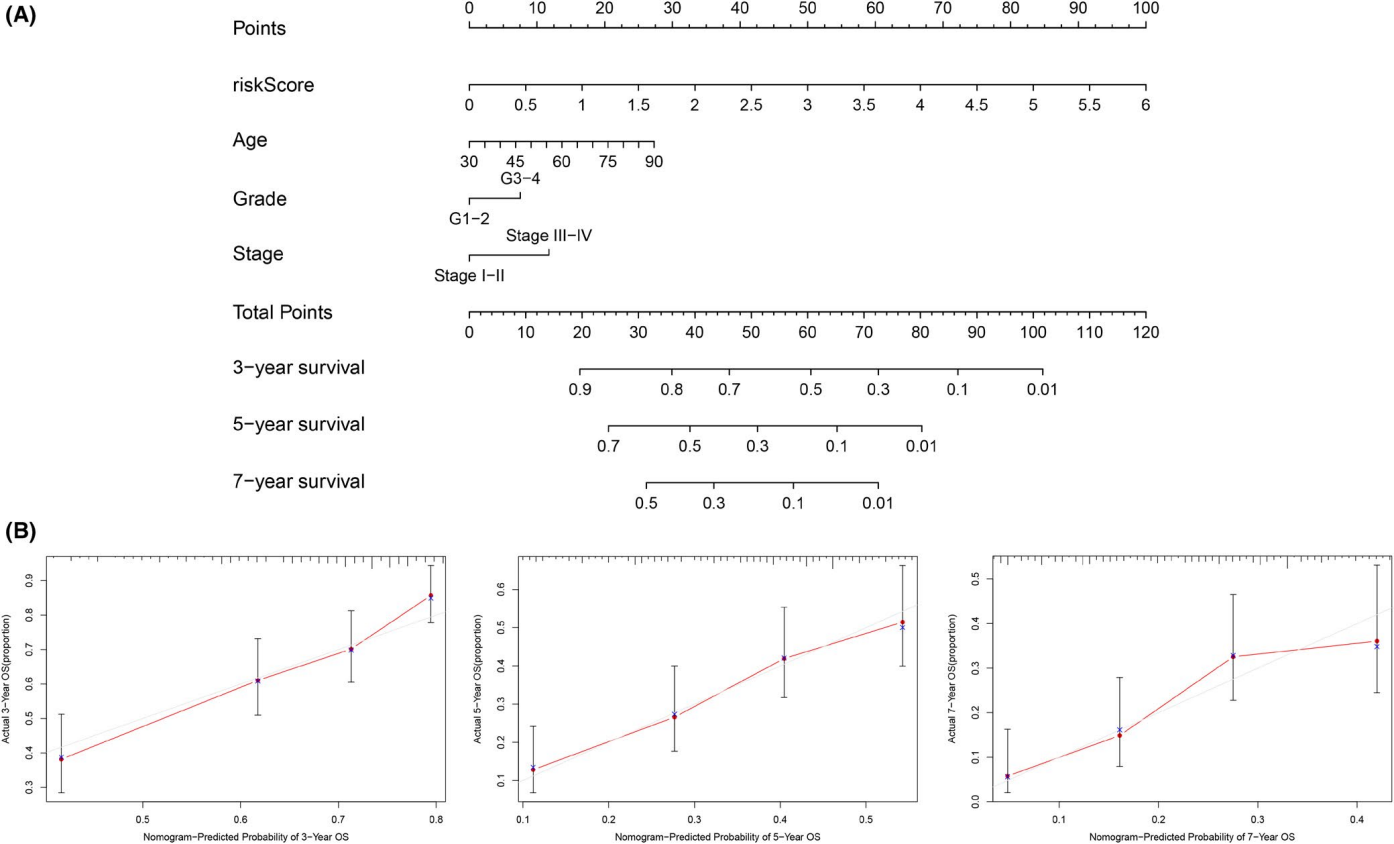
Establishment of the prognostic nomogram. (A) Nomogram for predicting 3‐, 5‐, and 7‐year overall survival of OC patients and (B) 3‐, 5‐, and 7‐year nomogram calibration curves of the prognostic nomogram

In order to provide clinicians with more convenient and accurate tools, we use clinical characteristics and risk scores to construct a nomogram^23^ (Figure 9A). On the calibration plots, a good correlation was observed between the predicted and the actual values of the OS rate of OC patients at 3, 5, and 7 years (Figure 9B).

### 3.8 Correlation analysis between the eight‐mRNA signature and immune cells

**FIGURE 10:**
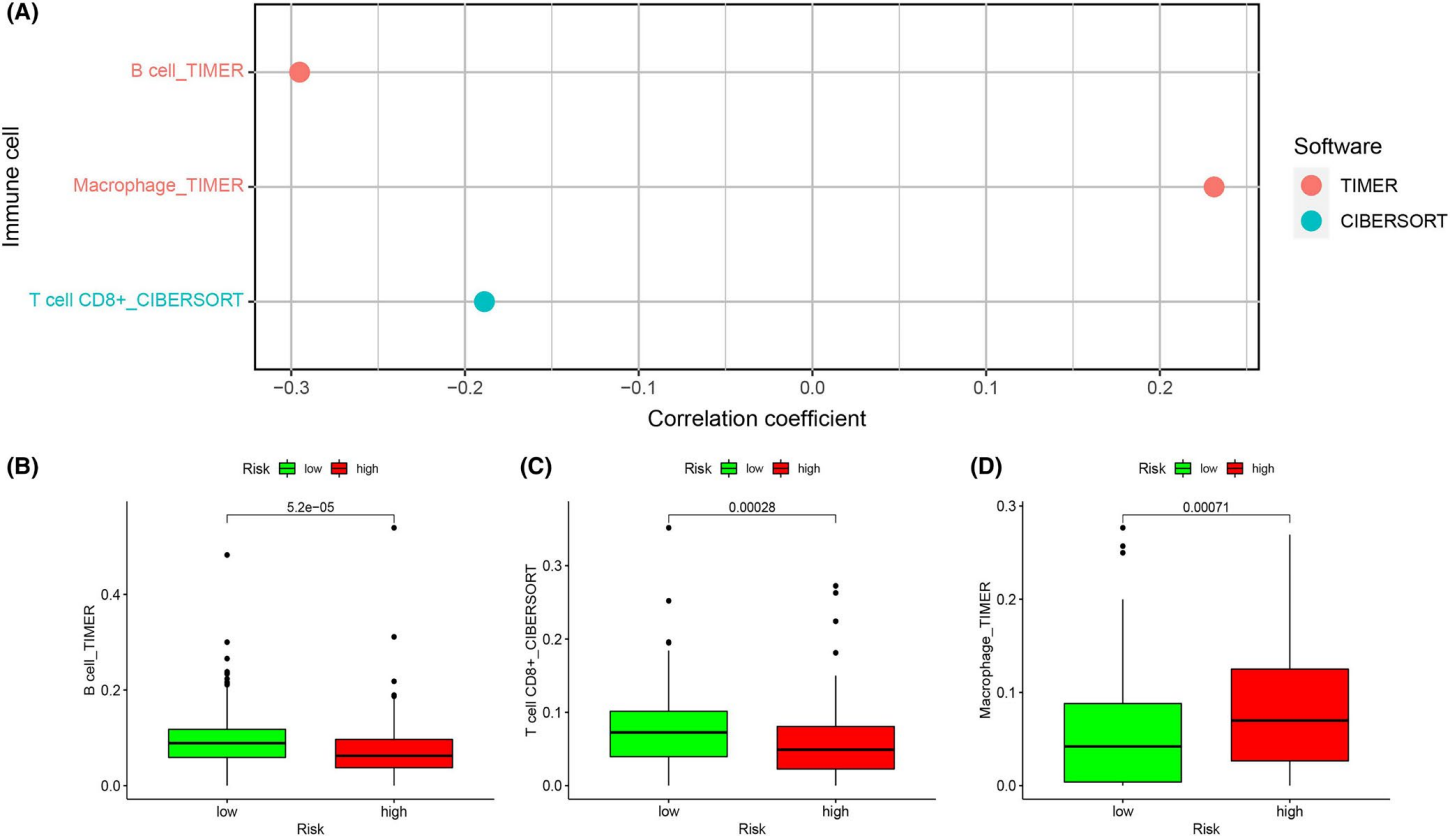
Correlation analysis of risk value and immune cells. (A) Correlation coefficient between eight‐mRNA signature and immune cells. (B) The expression of B cell in high‐ and low‐risk groups; (C) the expression of CD8+ T cell in high‐ and low‐risk patients; and (D) the expression of Macrophage in high‐ and low‐risk patients

We further identified the association between the aging‐related risk model and immune cells (Figure 10A). Using *p *< 0.05 as the filter criterion, we found that the risk score was associated negatively with B cells and CD8+ T cells, and the expression of B cells and CD8+ T cells was relatively high when the risk score was low (Figure 10B, *p* = 5.2e−05, Figure 10C, *p* = 0.00028). Macrophages were positively related to the risk score, and the expression of macrophages was higher in the high‐risk group (Figure 10D, *p* = 0.00071). CD8+ T cells are involved in the growth and functional maintenance of tumor.

### 3.9 Analysis of drug sensitivity related to the eight‐mRNA signature

**FIGURE 11:**
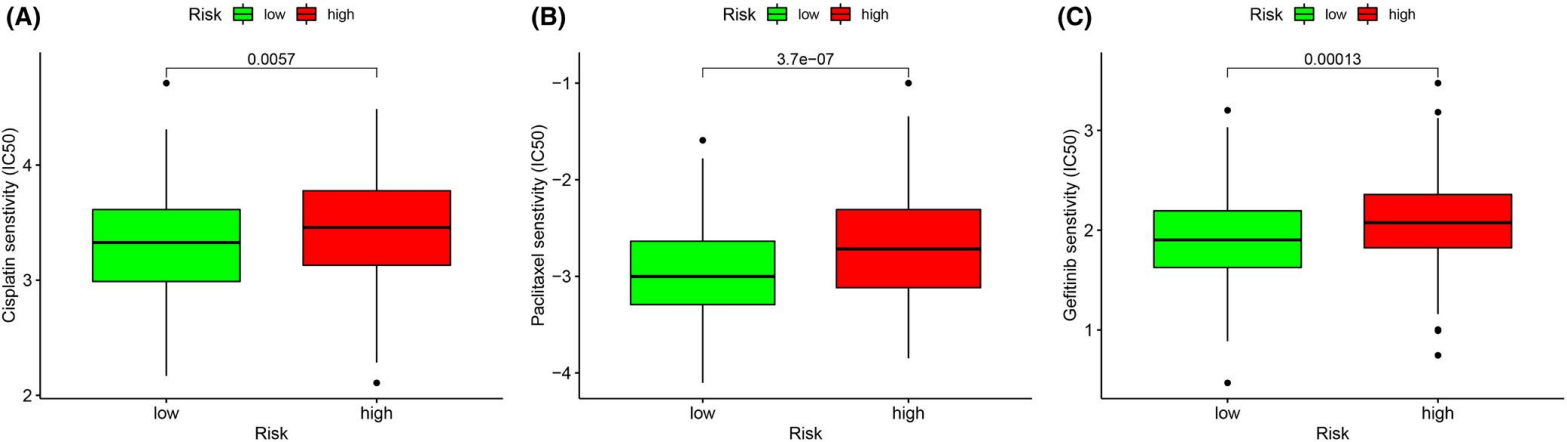
Drug sensitivity analysis. (A) Cisplatin, (B) Paclitaxel, and (C) Gefitinib

This study indicated that the high‐risk patients may respond better compared with the low‐risk patients treated with the same drugs (Figure 11A–C). Currently, platinum‐based chemotherapy is the cornerstone of the treatment for advanced OC. However, the existence of drug resistance and heterogeneity leads to differences in the therapeutic effects of drugs among different populations. This study revealed the sensitivity of high‐ and low‐risk patients to cisplatin, paclitaxel, and gefitinib, which will provide researchers with a new perspective on the development of drugs with higher efficacy. It also suggests that clinicians should intervene in the high‐risk group with corresponding drugs earlier, which may improve the survival rate of high‐risk patients, providing a new vision for future clinical work.

### 3.10 Validation of candidate genes by qPCR

**FIGURE 12:**
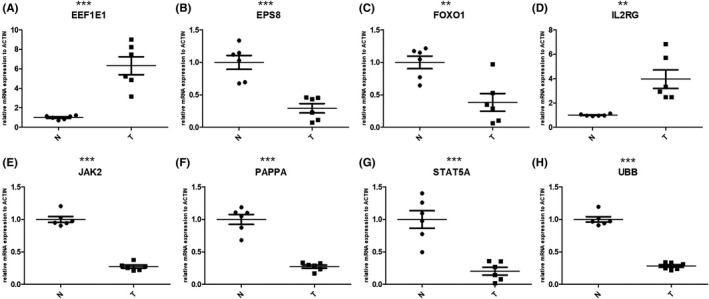
Validation of candidate genes by qPCR. (A) EEF1E1, (B) EPS8, (C) FOXO1, (D) IL2RG, (E) JAK2, (F) PAPPA, (G) STAT5A, and (H) UBB

We further examined the differential expression of JAK2, IL2RG, EEF1E1, UBB, EPS8, FOXO1, STAT5A, and PAPPA genes between OC and normal ovarian tissue samples. The RT‐qPCR results showed that compared to normal ovarian tissue samples, trends in the expression levels of these genes were consistent with our previous findings (Figure 12).

## 4 DISCUSSION

OC is the most fatal gynecological malignancy in women.^2^ Since age is one of the main reason for OC,^15^, ^16^, ^17^ with the aging process, the incidence of OC will continue to increase,^2^ and the process of aging and cancer is inseparable.^14^

Following the advent of new high‐throughput sequencing technology and the development of the TCGA and GEO databases, scholars have established a large number of risk models based on gene expression levels that can better predict the OS rate of OC patients. However, among the various risk characteristics, there is no aging‐related gene signature in predicting the outcome of OC patients.

In our study, we identified 285 AGs in the TCGA training group and GSE26712 validation group. After screening for differential genes, 113 DEAGs that were either upregulated or downregulated were found in the training group and validation group 1. After univariate Cox analysis, Lasso regression and multivariate Cox analysis of candidate genes in the training group, the aging‐related gene signature of eight genes was further constructed, including JAK2, IL2RG, EEF1E1, UBB, EPS8, FOXO1, STAT5A, and PAPPA. Following the comprehensive analysis of this signature, we observed that the risk score related to aging is notably correlated with the OS rate of OC patients. According to the area under the ROC curve, the prediction results were accurate, and we conducted external verification in three GEO data sets. Moreover, compared with other clinicopathological characteristics, the risk score signature showed a more reliable predictive ability. In addition, we constructed a nomogram combining risk score and age as a more convenient clinical tool for predicting prognosis. Additionally, the correlation between the level of the risk score and the infiltration of immune cells was analyzed, revealing that the risk score was positively correlated with macrophage and negatively correlated with CD8+ T cells and B cell. Finally, based on the risk score, we can judge the sensitivity of patients to paclitaxel, carboplatin, and gefitinib, allowing for timely treatment with the appropriate drugs.

At the present level of science and technology, aging is inevitable and is characterized by the stagnation of the cell cycle at the micro level and the gradual loss of function of tissues and organs at the macro level.^24^, ^25^ Cell senescence plays an important role in tumorigenesis, tumor development, and tumor immune escape. For example, the senescence‐associated secretory phenotype (SASP) is not only a tumor suppressor but can also act as a tumor driver.^24^ On the one hand, aging cells can induce senescence in adjacent tumor cells through autocrine and paracrine mechanisms by releasing SASP and activate immune surveillance to eliminate senescent and proliferating tumor cells at the same time, thereby inhibiting the proliferation of cancer cells.^26^ On the other hand, aging cells can reshape the tumor microenvironment through SASP, promote cell proliferation, and drive tumor angiogenesis, thus promoting tumor progression.^27^, ^28^, ^29^

Among the eight genes in the eight‐mRNA signature of this study, EPS8, FOXO1, STAT5A, and PAPPA were risk factors, and JAK2, IL2RG, EEF1E1, and UBB were protective factors.

EPS8 is the substrate of epidermal growth factor receptor (EGFR) kinase activity.^30^ Previously, scholars found that EPS8 was usually overexpressed in advanced thyroid cancer, pancreatic cancer, oral squamous cell carcinoma, and pituitary tumors.^31^, ^32^, ^33^, ^34^ In our study, compared with normal ovarian tissue, EPS8 showed low expression in ovarian cancer tissue, which may be related to the heterogeneity of the tumor. EPS8 binds to AbI1 through its SH3 domain, and AbI1 binds EPS8 and SOS1 together, thereby promoting the formation of a trimeric complex that activates Rac.^30^ Rac activity is required for the metastasis and colony‐forming ability of ovarian cancer cells.^35^ Therefore, the metastatic potential of ovarian cancer is closely related to the integrity of the SOS1/EPS8/ABI1 complex. In addition, some scholars have found that the presence of the SOS1/EPS8/ABI1 complex correlated well to the continuous epithelial–mesenchymal transition (EMT) characteristics of ovarian cancer cells, and an intact complex is required for this procedure.^36^ Therefore, silenced or low expression of EPS8 can reduce the migration and metastatic colonizing ability of ovarian cancer cells.^35^ Thus, relatively high expression of EPS8 is a risk factor for ovarian cancer, which is consistent with our research results. Regarding FOXO1, it has long been reported that FOXO1 expression is downregulated in cervical cancer, kidney cancer, breast cancer, prostate cancer, endometrial cancer, and ovarian cancer.^37^, ^38^, ^39^, ^40^, ^41^, ^42^ In ovarian cancer, the progesterone receptor (PR‐B) induces cell senescence through FOXO1, and one of the characteristics of aging cells is the upregulation of FOXO1 expression.^43^ In our study, we reached the same conclusion that the expression level of FOXO1 is generally downregulated, while the expression level of FOXO1 is relatively higher in high‐risk patients, which indicates that patients in the high‐risk group are more prone to cell aging. STAT5A is an oncogene. Members of the STAT family are related to the occurrence, progression, metastasis, angiogenesis, and immune escape of human cancer.^44^ In prostate cancer, some scholars have found that knocking down STAT5A can increase the sensitivity of prostate cancer to radiotherapy and reduce radiation damage to adjacent tissues^45^; in colorectal cancer, inhibition of STAT5A promotes chemotherapy (such as cisplatin or 5‐FU)‐induced apoptosis of colorectal cancer cells.^46^ In terms of immunity, STAT5 plays a key role in the function and development of Tregs, and continuously activated STAT5 can inhibit antitumor immunity and increase the proliferation, invasion, and survival of tumor cells.^47^ In our study, STAT5 was a risk factor, and relatively high expression of STAT5 increased the risk score and predicted a poor prognosis. Regarding PAPPA, although we did not find a differential level of PAPP‐A expression between normal ovarian tissue and ovarian cancer, there is evidence that the downregulation of the pregnancy‐associated plasma protein A (PAPPA) gene can reduce IGF‐I‐dependent Akt and ERK1/2 activation, thereby reducing the growth, invasion, and metastasis of OC cells.^48^ Similarly, it has been found that overexpression of PAPPA in ovarian cancer cells promotes the growth of tumors.^49^ According to our results, PAPPA is a risk factor, and the relatively high expression of PAPPA may be one of the reasons for poor prognosis in the high‐risk group.

Among the protective factors, JAK2 expression has been found to be upregulated in OC tissues after paclitaxel chemotherapy, and its expression is related to the drug resistance mechanism of OC.^50^ The JAK2‐STAT3 pathway promotes the development of paclitaxel resistance by upregulating the expression of pro‐survival and anti‐apoptosis genes. Inhibition of JAK2 can reverse the resistance of ovarian cancer to paclitaxel.^51^ Compared with normal tissues, the expression of JAK2 in OC is downregulated, which may be related to the heterogeneity of the tumor. In tumor tissues, the expression of JAK2 is relatively higher in low‐risk patients, which may be caused by chemotherapy and resistance. This point is worthy of further study. Regarding EEF1E1, studies have found that EEF1E1 is overexpressed in most tumors, including ovarian cancer, and that high expression of EEF1G predicts better OS and PFS rates in OC patients,^52^ which is consistent with our research results. In addition, in our study, UBB was a protective factor, and the downregulation of UBB predicted a worse prognosis, which is consistent with the conclusion of other studies. In human gynecological cancer, the expression of UBB is decreased, and the low expression of UBB is associated with the poor survival rate of gynecological cancer.^53^

Although age is an important risk factor for the development of OC, there are few published data to demonstrate the influence of aging and aging genes on OC. Only in animal studies has it been found that aging increases susceptibility to ovarian cancer metastasis in a mouse allograft model.^54^ Therefore, our eight‐mRNA signature and nomogram can provide new perspectives for clinical work.

Interestingly, we also found some differences in immune cell infiltration between the high‐ and low‐risk groups, suggesting that aging‐related genes may be related to tumor immunity. Several previous studies have shed light on the relationship between aging and immunity. The characteristic of aging is graduate senescent immune remodeling termed immunosenescence. For example, the immunosenescence of T cells, including shrinkage of immune repertoire, the exhaustion of memory T cells, and the reduction of immune costimulatory molecules, all of them lead to a significant decline in immune function with aging. One of the main tasks of the human immune system is cancer detection. Mutations or genetic disorders in somatic cells may cause them to be considered foreign antigens. For the elderly, as the age increases, the decline of immune function will reduce the recognition and elimination of these cells, eventually leading to their accumulation.^55^ These senescent cells and SASP factors can reprogram the tumor microenvironment into an environment more prone to the growth of malignant cells, which has been proven by a large number of studies.^56^, ^57^, ^58^, ^59^ In our study, the expression levels of B cells and CD8+T cells were significantly higher in the low‐risk group. As far as we know, CD8+T cells play a pivotal role in controlling tumor cell growth, and prior studies have already demonstrated that CD8+ T cells indicate a better prognosis.^60^ Other studies have found that not only does the percentage of peripheral blood B cells decrease with the increase in age, but also their capacity to spontaneously secrete IgM decreases.^61^ In contrast, the expression of macrophages was higher in the high‐risk group. Some scholars have observed an increase in the number of macrophages in aging adipose tissue; in addition, the increase in macrophages in the lymphatic tissues of elderly individuals was dominated by an increase in immunosuppressive M2 macrophages.^62^ The above may be one of the reasons for the better prognosis of the low‐risk group.

Platinum‐based chemotherapy is the first‐line chemotherapy for ovarian cancer. Given its limited specificity, many of the side effects associated with the treatment tend to endanger the lives of more elderly people. There are many examples of how the aging microenvironment can lead to chemotherapy resistance. Senescent fibroblasts with SASP and other stromal components secrete cytokines that promote cancer cell resistance to chemotherapy in a paracrine manner.^63^, ^64^, ^65^ In addition, research supports the fact that chemotherapy can further induce SASP in tumor, immune, and stromal cells through treatment‐induced senescent cells. Therefore, based on our sensitivity analysis, we may be able to draw a conclusion: high‐risk populations were more sensitive to platinum‐based chemotherapeutics, but due to the development of SASP, they developed drug resistance faster, and finally led to a poor treatment outcome. However, as we did not know whether the population included in the study had previously experienced platinum‐based treatment, this conclusion requires further exploration.

Although we have made many efforts to study the prognostic model, there are still some shortcomings. First, the ROC value of the external validation groups was unsatisfactory. Second, some clinical features such as postoperative interventions, radiotherapy, and chemotherapy for OC patients extracted from the TCGA and GEO databases are incomplete and not available, so at the time of writing, we could not conduct a full analysis of OS. Third, because clinical samples are relatively difficult to obtain, we only used six pairs of clinical samples to verify our conclusion. Currently we are actively collecting more clinical samples, and multicenter, large‐scale prospective, and well‐designed studies are required to further verify the prediction model presented here.

## 5 CONCLUSION

All in all, we constructed a prognostic signature of eight aging‐related genes and a clinical nomogram that provides potential biomarkers for predicting the prognosis of patients with OC, helps to understand the potential pathogenesis of OC, and can possibly be used to develop new approaches for the clinical treatment of ovarian cancer.

## ETHICS STATEMENT

Ethical approval for this study was obtained from the Ethics Committee of the Wenzhou Medical University. Clinical research ethics review YS2020‐218. And written informed consent was obtained from all patients.

## CONFLICT OF INTEREST STATEMENT

The author has no conflict of interest to declare.

## AUTHOR CONTRIBUTION

The study was conceived and designed by XJY and WFL. The manuscript was written by LXL and JDZ. XDD and YZ revised and edited the manuscript. HLZ and CYZ provided support for picture processing. The final version of the manuscript has been read and approved by all authors prior to submission.

## Supporting information

Fig S1

Table S1

## Data Availability

We agree to share data when this is applicable.
