# Supplementary figures and images for: Construction and validation of a novel aging‐related gene signature and prognostic nomogram for predicting the overall survival in ovarian cancer
Source: Cancer Med. 2021 Nov 25;10(24):9097–114. doi: 10.1002/cam4.4404 (PMC8683552; doi:10.1002/cam4.4404)

(A)

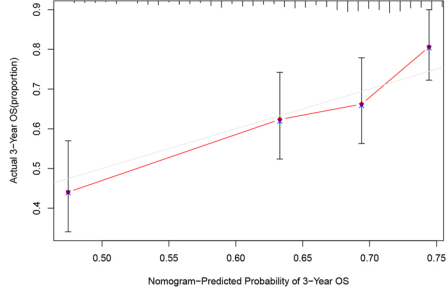

(B)

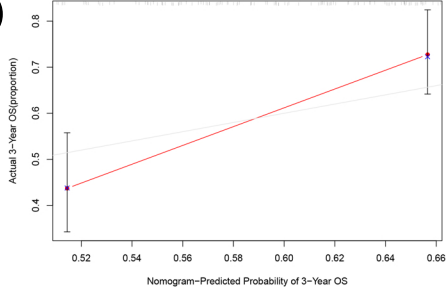

(C)

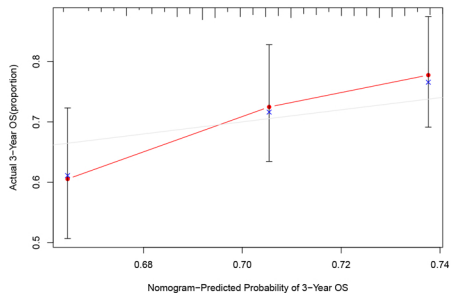

(D)

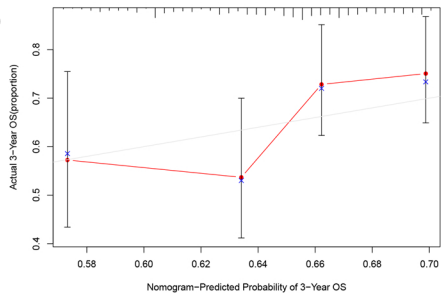

Supplement: Supplementary file 1 — Fig S1 [file CAM4-10-9097-s002.pdf]
